# Molecular Evidence of Drug-Resistant Tuberculosis in the Balimo Region of Papua New Guinea

**DOI:** 10.3390/tropicalmed4010033

**Published:** 2019-02-10

**Authors:** Tanya Diefenbach-Elstob, Vanina Guernier, Graham Burgess, Daniel Pelowa, Robert Dowi, Bisato Gula, Munish Puri, William Pomat, Emma McBryde, David Plummer, Catherine Rush, Jeffrey Warner

**Affiliations:** 1 College of Public Health, Medical and Veterinary Sciences, James Cook University, Townsville 4811, Australia; graham.burgess@jcu.edu.au (G.B.); munish.puri@my.jcu.edu.au (M.P.); david.plummer@jcu.edu.au (D.P.); catherine.rush@jcu.edu.au (C.R.); jeffrey.warner@jcu.edu.au (J.W.); 2 Australian Institute of Tropical Health and Medicine, James Cook University, Townsville 4811, Australia; vanina.guernier@gmail.com (V.G.); emma.mcbryde@jcu.edu.au (E.M.); 3 Balimo District Hospital, Balimo 336, Papua New Guinea; tanya.elstob@jcu.edu.au (D.P.); dowir733@gmail.com (R.D.); bisatokela01@gmail.com (B.G.); 4 Papua New Guinea Institute of Medical Research, Goroka 441, Papua New Guinea; william.pomat@pngimr.org.pg

**Keywords:** tuberculosis, *Mycobacterium tuberculosis*, drug resistance, real-time PCR

## Abstract

Papua New Guinea (PNG) has a high burden of tuberculosis (TB), including drug-resistant TB (DR-TB). DR-TB has been identified in patients in Western Province, although there has been limited study outside the provincial capital of Daru. This study focuses on the Balimo region of Western Province, aiming to identify the proportion of DR-TB, and characterise *Mycobacterium tuberculosis* (MTB) drug resistance-associated gene mutations. Sputum samples were investigated for MTB infection using published molecular methods. DNA from MTB-positive samples was amplified and sequenced, targeting the *rpoB* and *katG* genes to identify mutations associated with rifampicin and isoniazid resistance respectively. A total of 240 sputum samples were collected at Balimo District Hospital (BDH). Of these, 86 were classified as positive based on the results of the molecular assays. For samples where *rpoB* sequencing was successful, 10.0% (5/50, 95% CI 4.4–21.4%) were considered rifampicin-resistant through detection of drug resistance-associated mutations. We have identified high rates of presumptive DR-TB in the Balimo region of Western Province, PNG. These results emphasise the importance of further surveillance, and strengthening of diagnostic and treatment services at BDH and throughout Western Province, to facilitate detection and treatment of DR-TB, and limit transmission in this setting.

## 1. Introduction

Papua New Guinea (PNG) is considered to have a high burden of tuberculosis (TB) and drug-resistant TB (DR-TB) [1]. In 2017, the estimated incidence of TB was 432 cases per 100,000 people nationally, while in 2016 the case notification rate for Western Province was 674 per 100,000 people [1,2]. Despite the high burden of TB, HIV is not considered to be a main driver of TB in Western Province [3]. In 2017, the national proportion of DR-TB was 3.4% of newly diagnosed TB cases, and 26% of previously treated cases, with this proportion including both rifampicin (RIF)-resistant TB (RR-TB) and multidrug-resistant TB (MDR-TB) (resistance to both RIF and isoniazid (INH)) [1].

Studies undertaken in PNG have identified MDR-TB at sites in Eastern Highlands, Gulf, Madang, Milne Bay, Morobe, and Western provinces, and the National Capital District [4,5,6,7,8]. At Daru Hospital in the provincial capital of Western Province, MDR-TB has been reported in 34.2% of new and previously treated cases, and extensively drug-resistant TB (MDR-TB with additional fluoroquinolone and second-line injectable antibiotic resistance) has also been described [3,4,9,10,11,12,13]. Other studies have found MDR-TB in 25% of Western Province-based TB patients presenting at Australian health clinics in the Torres Strait [14,15]. However, no previous research has investigated DR-TB in the Middle Fly District of Western Province, and the geographic origin of DR-TB patient samples referred to Daru Hospital (the referral hospital for DR-TB in the province) has not been reported.

Phenotypic drug susceptibility testing (DST) of *Mycobacterium tuberculosis* (MTB) bacilli isolated and cultured from patient clinical samples serves as a reference standard for the diagnosis of DR-TB [16]. However, culture-independent molecular methods have advantages over DST due to rapid turnaround time, and the ability to be used outside high-containment laboratories.

The molecular basis of RIF resistance has been well-characterised, with 96% of this resistance associated with mutations in an 81-base-pair (bp) hypervariable RIF-resistance determining region (RRDR) in the *rpoB* gene [17,18]. In addition, resistance to INH has been associated with mutations in the *katG* and *inhA* genes [17,19]. Because a large proportion of RIF-resistant strains have concomitant INH resistance, molecular detection of RIF resistance is often used as an early indicator of MDR-TB before phenotypic susceptibilities are available, or in countries where DST is not routinely available [20]. However, the pattern and frequency of mutations in the *rpoB* and *katG* genes in MTB clinical isolates have significant geographic variability [21,22]. As a result, techniques that focus only on *rpoB,* such as the World Health Organization (WHO)-recommended Xpert MTB/RIF (Cepheid, USA), may overestimate MDR-TB, especially if the local prevalence of RR-TB is unknown. Therefore, an approach that can identify both *rpoB* and *katG* mutations is more likely to differentiate RR-TB, MDR-TB, and strains in which MDR-TB is likely to develop (pre-MDR-TB) [23].

The Balimo region in the Middle Fly District of Western Province is known to have a high burden of TB [24], with limited diagnostic facilities and smear microscopy being the only laboratory-based method of TB diagnosis available at Balimo District Hospital (BDH). Given the TB epidemic in Western Province, combined with limited data from areas outside the provincial capital, the aim of this study was to characterise the extent and type of molecular DR-TB in the remote Balimo region. As a preliminary study into DR-TB in this region, where techniques such as the Xpert MTB/RIF are not available, we used a pragmatic approach focused on the characterisation of RIF and INH resistance, based on mutations in the *rpoB* and *katG* genes. We characterised molecular evidence of resistance using DNA extracted directly from sputum samples collected at BDH. This study furthers understanding of the burden of DR-TB in Western Province.

## 2. Study Population and Methods

### 2.1. Study Setting

Balimo is a town of approximately 4400 people and is the urban centre of the Gogodala Rural local level government area in the Middle Fly District of Western Province [25]. Numerous small villages make up the Gogodala region, with a population of approximately 33,000 people [25]. The region is geographically remote with limited road networks, and travel is primarily by boat or by foot. Income is limited, with most people having subsistence-based livelihoods.

The primary health facility serving the region is BDH. However, the hospital has been without a physician for many years, and staffed by health extension officers and nurses. At the hospital, diagnosis of TB relies on clinical presentation and smear microscopy results, and is based on the PNG National Tuberculosis Management Protocol [24,26,27]. The hospital laboratory does not have the capacity for culture or Xpert MTB/RIF-based diagnosis of TB. Patients can be commenced on treatment for drug-susceptible TB, while presumptive DR-TB cases must be referred to Daru Hospital. For this study, sputum samples were collected from presumptive TB patients as part of routine passive case detection activities at BDH. Associated demographic and clinical data were obtained from the BDH laboratory register, however information about the TB treatment history of patients was not available from this register.

### 2.2. Initial Collection and Preparation of Samples

Sputum samples were collected during the period from April 2016 to June 2017, as part of ongoing passive case detection of TB as per the BDH clinical procedures. A total of 240 samples were provided on a convenience sampling basis for this study. Processed sputum (*n* = 213) was prepared at the BDH laboratory, using decontamination and concentration procedures according to the modified Petroff’s method [28,29], and stored at −20 °C. Following processing, sputum was smeared onto microscope slides, and examined for the presence of acid-fast bacilli using Ziehl-Neelsen (ZN) staining. Fresh sputum samples (*n* = 27) were collected in the few days prior to transfer of the samples to Townsville. For this reason, the fresh sputum samples could not be processed at BDH as usual, and instead were decontaminated and concentrated at James Cook University, Townsville, following the same procedure used at BDH.

### 2.3. Preparation of DNA Template

Approximately 100–150 µL of each decontaminated sputum was transferred to a 2 mL tube and heat inactivated at 80 °C in a thermal block for 1 h. Sputum was then centrifuged at 3000× *g* for 15 min, and supernatant removed. A volume of 1 mL of a 4 M guanidine isothiocyanate (GIT) solution was added to each tube, and incubated overnight at 37 °C. Samples were then centrifuged at 13,000 rpm for 10 min, and supernatant removed.

Following inactivation, DNA was extracted using the High Pure PCR Template Preparation Kit (Roche Diagnostics, Germany). The spun pellets were resuspended in 200 µL of tissue lysis buffer, and 40 µL of proteinase K solution, and incubated overnight at 55 °C, or until all sediment was dissolved. Subsequent steps were performed according to the manufacturer’s instructions. Extracted DNA was stored at −80 °C until use.

### 2.4. Confirmation of MTBC Infection

Two TaqMan real-time polymerase chain reaction (qPCR) assays were used to confirm the presence of the *Mycobacterium* species (IS*6110* assay) or *M. tuberculosis* complex (MTBC) (*senX3-regX3* assay) in the DNA extracts, according to published protocols [30]. While the *senX3-regX3* intergenic region is specific to the MTBC, IS*6110* is present in most strains of *M. tuberculosis,* but has also been identified in other species of *Mycobacterium*, including non-tuberculous mycobacteria (NTM). Both targets were used because the IS*6110* assay, which targets a multicopy element, is more sensitive than the *senX3-regX3* assay, which targets a single copy gene [30].

Each qPCR reaction (20 µL) contained 1X GoTaq Probe qPCR Master Mix (Promega, Madison, WI, USA), 0.8 µM each of forward and reverse primer, 0.1 µM of probe, and 2 µL of DNA template. The positive control used MTB H37Rv (GenBank: AL123456.3). Cycling parameters are shown in Table 1. qPCR assays were carried out on a Rotor-Gene Q6000 (QIAGEN, Hilden, Germany).

Each sample was assayed in duplicate for the IS*6110* qPCR, with a triplicate assay for discordant results, and assayed once for the *senX3-regX3* qPCR. A sample was considered reactive if the cycle threshold (Cq) was less than 40 cycles. Samples with duplicate reactivity of the IS*6110* assay as well as reactivity of the *senX3-regX3* assay were considered MTB-positive, while samples with duplicate reactivity of the IS*6110* assay only were classified as MTBC/NTM. In PNG, only a small number of NTM strains have been identified, and they are likely to have limited influence in the context of our study [4,26,31]. However, because the possibility of rare NTM in the IS*6110*-positive samples cannot be completely excluded, we have used the ‘MTBC/NTM’ classification for accuracy.

### 2.5. rpoB and katG Mutation Analysis

Targeted PCR was used to identify mutations in the *rpoB* and *katG* genes in DNA extracted from the TB clinical samples. Each PCR reaction (25 µL) contained 1X GoTaq G2 Green Master Mix (Promega, Madison, WI, USA), 0.5 (*rpoB*) or 0.8 (*katG*) µM each of forward and reverse primer, and 2 µL of DNA template. The positive control used MTB H37Rv (GenBank: AL123456.3). The *rpoB* and *katG* primer sets have been described elsewhere [8], with the published conditions optimised in-house for each protocol, as described in Table 1. The PCR assays were undertaken on BIOER GeneTouch and Kyratec SuperCycler Trinity PCR machines. Amplicons showing single clear bands after agarose gel electrophoresis were sequenced in both directions (Macrogen, Korea). Sequence chromatograms were analysed using Geneious R10 (Biomatters Limited, Auckland, New Zealand), with the consensus sequences of each sample compared to the MTB H37Rv reference sequence (GenBank: AL123456.3). The numbering system used in the results is according to that described previously for MTB H37Rv [32].

### 2.6. Analysis of Duplicate and Repeat Samples

Duplicate samples were excluded from the analyses for four patients. Three patients had both new (initial TB investigation) and follow-up (post-treatment commencement) samples collected and included in the analysis. Follow-up samples were collected at different time-points, ranging from two to four months following treatment commencement.

### 2.7. Ethics Approval

The study was undertaken in collaboration with BDH, and received approval locally from the Middle Fly District Health Service, and the Evangelical Church of PNG Health Service. Human research ethics approval was received from the James Cook University Human Research Ethics Committee (Ethics Approval Number H6432) and the PNG Medical Research Advisory Committee (MRAC No. 17.02).

## 3. Results

### 3.1. Demographic Information and Detection of MTB Infection

Overall, 240 sputum samples were collected, with 236 samples from 233 patients analysed based on the exclusion criteria described previously (see Methods). Patient distributions by age and sex are shown in Table 2. Sample status at time of collection, and smear microscopy results for new samples only, are shown in Table 3.

Based on the classification criteria for molecular detection of MTB (see Methods), a total of 62/236 (26%, 95% CI 21–32) samples were classified as MTB, and 24/236 (10%, 95% CI 7–15) were classified as MTBC/NTM. The remainder (150/236, 64%, 95% CI 57–69) were classified as negative for MTB. Of the 24 samples classified as MTBC/NTM, 22 had Cq values greater than 30 cycles (see Supplementary Material).

### 3.2. Mutations Identified in MTB DNA

Of the 240 sputum samples that were collected, 102 were assessed for resistance using the *rpoB* and *katG* primer sets. This included 87 samples classified as MTB (*n* = 62) or MTBC/NTM (*n* = 25), as well as 15 samples reactive for early runs of the IS*6110* assay or a *rpoB*-based assay being tested externally to this study, but ultimately classified as negative. One MTBC/NTM sample was excluded from the results due to being a duplicate (see Methods).

Amplicons were obtained from 53 of the 86 samples classified as MTB or MTBC/NTM. Overall, sequence data was obtained from one MTBC/NTM sample, while all other sequences were obtained from samples classified as MTB.

*rpoB* sequencing was successful for 50 samples (Table 4). One sample classified as MTB based on qPCR did not match the H37Rv reference strain, and thus may not have been MTB. RIF resistance-associated mutations were identified in five samples, as detailed in Table 4. These five samples were all classified as new (i.e., they were collected from people undergoing initial investigations for TB). Overall, 10% (5/50, 95% CI 4–21) of samples with *rpoB* sequencing were considered to be RIF-resistant, based on identification of a *rpoB* mutation that is known to confer drug resistance.

*katG* sequencing was successful for 47 samples, with mutations identified in two samples as described in Table 4. We were unable to confirm mutations in five samples because of discordant sequencing results in the forward and reverse strands. These included *rpoB* WT/F548L in one sample, *katG* WT/E261K in one sample, and *katG* WT/G279R in three samples (Table 4). These discordant results were not investigated further due to the high possibility of sequencing error, and as they have not previously been reported or associated with drug resistance their clinical significance is unknown.

## 4. Discussion

This study was undertaken on sputum samples collected in the rural Balimo region of PNG from presumptive TB patients. Molecular diagnostic techniques identified MTB in 26% to 36% (when including MTBC/NTM) of the samples. In our setting, classification as MTBC/NTM rather than MTB may have been due to a reduced ability to amplify the single copy *senX3-regX3* gene, as a result of low MTB DNA concentration. As such, classification of MTBC/NTM is not considered to exclude MTB, but is simply a lack of confirmation. DR-TB was identified in samples collected at BDH, with RIF resistance-associated mutations identified in 10% (5/50, 95% CI 4–21) of the MTB or MTBC/NTM samples where *rpoB* sequencing was obtained. 

The *rpoB* S450L codon mutation, identified in five samples in this study, is the most frequently identified RIF resistance-associated mutation in the *rpoB* gene [33,34]. This mutation has been described previously in three studies from PNG, including in Western Province [5,8,13]. The *rpoB* I480V codon mutation, identified in one sample in this study, is much less common, and was first described in a study from Mexico, where it appeared alongside S450L as in our sample [35]. Interestingly, the same double amino acid change has recently been identified in a single clinical sample collected at Daru Hospital in Western Province, PNG [13]. However, the geographic origin of this patient was not stated.

There is less certainty regarding the INH drug resistance association of the mutations identified in the *katG* gene. In this study S315T, the most common INH resistance-associated *katG* mutation, was not identified, despite being seen in other studies from PNG, including in Western Province [5,8,13]. However, *katG* mutations were identified in a number of sequences, and these should be investigated further to confirm the possible presence of novel mutations, as well as for phenotypic DST. In addition, the *inhA* and *ahpC* genes, as well as other genes that have been associated with INH resistance, were not investigated. As a result, neither INH mono-resistance nor MDR-TB could be determined. Despite this finding, genotypic INH resistance should continue to be monitored, as several hundred different *katG* mutations have been documented in INH-resistant TB samples [19,36,37], with descriptions of new mutations likely to occur in the future. Additionally, *inhA* mutations have been identified in INH-resistant MTB strains elsewhere in PNG, including in Western Province [5,8,13].

For this study, repeat sequencing would be necessary to confirm mutations that have not previously been associated with drug resistance, and culture and DST would be required to confirm the phenotypic drug resistance status of *rpoB* and *katG* mutations identified, especially given some mutations may be seen in both drug-susceptible and drug-resistant isolates [19,36,38,39].

We identified RIF resistance-associated mutations in 10% (95% CI 4–21) of samples where *rpoB* sequencing was successful, indicating that DR-TB is already an established concern in the Middle Fly District. As described earlier, a high proportion of DR-TB has been described at Daru Hospital in the South Fly District of Western Province [4]. However, as Daru Hospital is the site for Xpert MTB/RIF testing of samples from across Western Province [27], clinical samples tested there will have originated from local residents as well as patients from elsewhere in the province, including Balimo. As a result, the proportion of DR-TB identified in Daru would be expected to be higher than the national average. The earlier study describing DR-TB in Daru, in combination with the results of our study undertaken in Balimo, highlight the geographic reach of DR-TB across much of Western Province. 

This was a small study, undertaken on samples collected on a passive case detection basis at BDH. The study was a laboratory-based analysis of samples collected as part of passive case detection activities at BDH, and the TB treatment history of patients was not recorded in the laboratory register. Furthermore, sequencing results were not obtained for 33 of the samples classified as MTB or MTBC/NTM. A larger study would be necessary to provide greater understanding of the epidemiology of DR-TB in the Balimo region. Further investigation would be particularly useful in understanding the treatment history and geographic distribution of DR-TB patients, and the genetic diversity of DR-TB strains.

## 5. Conclusions

This study has described the presence of RR-TB in the Balimo region, based on the identification of resistance-associated mutations in the *rpoB* gene. These findings extend our earlier research in Balimo, which has described a heavy burden of TB in the region, and highlighted the potential underdiagnosis of smear-negative pulmonary TB at BDH [24,26]. Identification of RR-TB in the Balimo region emphasises the need for further investigation of the DR-TB burden in the Middle Fly District of Western Province. Analysis of the TB treatment history of DR-TB patients would provide insight into the contribution of the various pathways to DR-TB in this region, that is, development versus transmission. Implementation in Balimo of a method such as the WHO-recommended Xpert MTB/RIF, which is capable of TB diagnosis and detection of RIF resistance, should be considered. Currently in Balimo, clinical samples from presumptive DR-TB patients must be sent to the provincial capital of Daru for Xpert MTB/RIF testing [27]. If the sample is positive, the patient must then travel to Daru to be commenced on DR-TB treatment, with completion under the supervision of an appropriately trained health worker [27]. Potential patient and provider challenges associated with this approach include delays in initiation of adequate treatment, active transmission of DR-TB to others, loss-to-follow-up while waiting for diagnostic results, and access challenges due to the long travel time to reach Daru. The presence of RIF resistance in this region distant from Daru emphasises the importance of understanding the heterogeneity of DR-TB across Western Province. Improving resources and facilities at BDH for local management of TB and DR-TB patients may reduce the strain placed on Daru Hospital, and play a role in the management of DR-TB patients in Western Province.

## Figures and Tables

**Table 1:** Cycling parameters for the real-time PCR (qPCR) and conventional PCR (*rpoB* and *katG*) assays.

Assay	No. Cycles	Thermal Conditions
IS*6110*/*senX3-regX3*	1	95 °C for 2 min
	45	95 °C for 5 s
		60 °C for 15 s
*rpoB*	1	94 °C for 2 min
	35	94 °C for 30 s
		58 °C for 30 s
		72 °C for 40 s
	1	72 °C for 5 min
*katG*	1	94 °C for 2 min
	40	94 °C for 30 s
		60 °C for 30 s
		72 °C for 60 s
	1	72 °C for 5 min

C: Celsius; No.: number.

**Table 2:** Demographics of presumptive tuberculosis (TB) patients in the Balimo region of Papua New Guinea (PNG), from samples collected as part of routine passive case detection activities at Balimo District Hospital (BDH). Out of 240 samples initially collected, a total of 236 samples originating from 233 patients were analysed, as three patients had both new and follow-up samples collected, and four samples were duplicates.

Category	-	*n* (%)
Sex	Female	123 (53)
	Male	109 (47)
	Unknown	1 (0.4)
	Total	233 (100)
Age	Child (0–17 years)	16 (7)
	Adult (18+ years)	216 (93)
	Unknown	1 (0.4)
	Total	233 (100)

*n*: number.

**Table 3:** Distribution of incoming sample status of all presumptive TB patients, and smear microscopy results for new samples only.

Category	-	*n* (%)
Sample status	New	211 (89)
	Follow-up	21 (9)
	Unknown	4 (2)
	Total	236 (100)
Smear microscopy result	Positive	32 (15)
	Negative	176 (83)
	Unknown	3 (1)
	Total	211 (100)

*n*: number.

**Table 4:** Summary of sequencing results for the *rpoB* and *katG* amplicons, including nucleotide and codon mutations.

*rpoB* Result	*katG* Result	Combined	*n*
WT	WT	WT	34
WT	N/A	*rpoB* WT	5
S450L ^1^ (C1349T)	WT	RIF-DR	4
		*katG* WT	
S450L ^1^ (C1349T)	WT	RIF-DR	1
I480V (A1438G)		*katG* WT	
WT or F548L (C1644A)	WT	*rpoB* discordant	1
		*katG* WT	
N/A	WT	*katG* WT	1
N/A	P219L ^2^ (C656T)	*katG* mutant	1
N/A	A361V ^2^ (C1082T)	*katG* mutant	1
	P365S ^2^ (C1093T)		
	S383L ^2^ (C1148T)		
	R396C ^2^ (C1186T)		
WT	WT or E261K ^2^ (G781A)	*rpoB* WT	1
		*katG* discordant	
WT	WT or G279R ^2^ (G835C)	*rpoB* WT	3
		*katG* discordant	
Not matched to ref	N/A	Not matched to ref	1
Total			53

*n*: number; N/A: not available (amplification or sequencing failed); ref: H37Rv reference genome; RIF-DR: rifampicin drug resistance; WT: wild-type (no mutations identified). ^1^ Codon mutation located within the RRDR of the *rpoB* gene, numbered according to the system based on MTB H37Rv [32]; ^2^ *katG* mutations with unknown association with drug resistance

## Supplementary Materials

The following are available online at https://www.mdpi.com/2414-6366/4/1/33/s1, Table S1: Details of qPCR and sequencing results.
